# Structural studies reveal a ring-shaped architecture of deep-sea vent phage NrS-1 polymerase

**DOI:** 10.1093/nar/gkaa071

**Published:** 2020-02-04

**Authors:** Xi Chen, Shichen Su, Yiqing Chen, Yanqing Gao, Yangyang Li, Zhiwei Shao, Yixi Zhang, Qiyuan Shao, Hehua Liu, Jixi Li, Jinbiao Ma, Jianhua Gan

**Affiliations:** 1 State Key Laboratory of Genetic Engineering, Collaborative Innovation Center of Genetics and Development, Shanghai Public Health Clinical Center, School of Life Sciences, Fudan University, Shanghai 200438, China; 2 State Key Laboratory of Genetic Engineering, Collaborative Innovation Center of Genetics and Development, Department of Biochemistry, School of Life Sciences, Fudan University, Shanghai 200438, China; 3 State Key Laboratory of Genetic Engineering, Collaborative Innovation Center of Genetics and Development, Department of Physiology and Biophysics, School of Life Sciences, Fudan University, Shanghai 200438, China

## Abstract

NrS-1 is the first known phage that can infect *Epsilonproteobacteria*, one of the predominant primary producers in the deep-sea hydrothermal vent ecosystems. NrS-1 polymerase is a multidomain enzyme and is one key component of the phage replisome. The N-terminal Prim/Pol and HBD domains are responsible for DNA polymerization and *de novo* primer synthesis activities of NrS-1 polymerase. However, the structure and function of the C-terminus (CTR) of NrS-1 polymerase are poorly understood. Here, we report two crystal structures, showing that NrS-1 CTR adopts one unique hexameric ring-shaped conformation. Although the central helicase domain of NrS-1 CTR shares structural similarity with the superfamily III helicases, the folds of the Head and Tail domains are completely novel. Via mutagenesis and *in vitro* biochemical analysis, we identified many residues important for the helicase and polymerization activities of NrS-1 polymerase. In addition to NrS-1 polymerase, our study may also help us identify and understand the functions of multidomain polymerases expressed by many NrS-1 related phages.

## INTRODUCTION

DNA polymerases are a group of enzymes that can catalyze DNA synthesis both *in vivo* and *in vitro*. Although they all share one conserved catalytic core, DNA polymerases from different subfamilies, such as A, B, X, Y and RT, show significant divergences on overall sequences and structures (1). The structural differences provide the basis for diverse functions played by DNA polymerases, including DNA replication (2,3), DNA repair (4) and translesion (5,6), which are all critical for genetic information maintenance in living cells. Interestingly, these common DNA polymerases are incapable of *de novo* DNA synthesis. To efficiently initiate the DNA synthesis process, one preexisting primer is required. Primers are usually synthesized by primases, a special group of polymerases that mainly function in DNA replication or repair initiation process (7,8).

Recently, a novel class of DNA polymerases (termed Primase-Polymerases, PrimPols) have been identified in humans (9,10) and archaea (11). Besides DNA polymerization, PrimPols also possess primer synthesis activities. However, different from regular primases that use ribonucleotides in primer synthesis, PrimPols start DNA chains with deoxyribonucleotides. Human PrimPol (*Hs*PrimPol) is present in both nuclear and mitochondrial compartments. *Hs*PrimPol has emerged as a novel DNA damage tolerance enzyme, it can bypass various DNA lesions (including 8oxoG and abasic site) during the DNA translesion process (10,12–14). In archaea, PrimPols are encoded by the extrachromosomal plasmids and responsible for the replication of the entire plasmid DNAs (15,16).

As the founding members of PrimPols, the pRN1 protein from *Sulfolobus islandicus* and *Hs*PrimPol have been extensively studied (17,18). The catalytic cores of pRN1 and *Hs*PrimPol are all located in the N-terminal regions. However, the overall sequences and domain compositions of the two proteins are very different. Following the catalytic core, pRN1 contains one small helix bundle domain (HBD), which binds the DNA template and prepares pRN1 for primer synthesis (19,20). At the C-terminus, pRN1 has one predicted helicase domain, which belongs to the D5_N superfamily. Unlike pRN1, *Hs*PrimPol contains one zinc finger module (Znf) at its C-terminus; this Znf module can bind to single-strand DNA (ssDNA) and is required for the primase activity of *Hs*PrimPol (13).

Inspired by the important functions of *Hs*PrimPol and pRN1, scientists have analyzed the genomes of many organisms in the past few years. Similar to archaea and humans, the PrimPol-coding genes were also found in bacteria and bacteriophage, suggesting that *de novo* DNA synthesis process is conserved across all the three kingdoms of life. In 2017, Zhu *et al.* characterized one very special DNA polymerase from deep-sea vent phage NrS-1 (21). NrS-1 is the first known phage that can infect *Epislonproteobacteria*, one predominant primary producer in deep-sea hydrothermal vent ecosystems (22). Like *Hs*PrimPol and pRN1, *in vitro* catalytic assays showed that NrS-1 polymerase is one self-priming polymerase and can synthesize long DNA strand at the absence of primer. However, different from *Hs*PrimPol and pRN1, which have very minimal template sequence requirement, the primer synthesis activity of NrS-1 polymerase is largely dependent on one 8-nt recognition sequence on the template.

The structures of the N-terminal region (residues 1–300, which was referred to as N300 hereafter) of NrS-1 polymerase have been reported (23). Structure and sequence analysis confirmed that NrS-1 polymerase shares conserved catalytic core with pRN1, *Hs*PrimPol and archaeal primases, such as *Pfu*Primase. Like pRN1, NrS-1 polymerase also contains one HBD domain at its N-terminal region, which is critical for the primer synthesis activity of NrS-1 polymerase. NrS-1 polymerase is one key component of the replisome responsible for NrS-1 genome replication. Based on *in vitro* biochemical analysis, it was suggested that the C-terminal region (CTR) of NrS-1 polymerase is essential for the proper function of NrS-1 replisome. However, the detailed structure and function of NrS-1 CTR have not been well investigated.

Herein, we report the structural and functional studies of NrS-1 polymerase. Totally, two NrS-1 CTR structures were determined, one in A-form and another in B-form. NrS-1 CTR assembles into ring-shaped hexamer in both structures. Although the central region (residues 404–593) shares sequence and structural similarities with AAA+ helicase domain, the Head (residues 304–391) and Tail (residues 619–718) regions are completely novel. Electron micrographic data analysis confirmed that the unique hexameric conformation can also be adopted by the full-length NrS-1 polymerase. In combination with mutagenesis, *in vitro* helicase and polymerization assays, our structures revealed many residues important for the function of NrS-1 polymerase.

## MATERIALS AND METHODS

### Plasmid construction

The gene containing the codon-optimized cDNA sequence of NrS-1 polymerase (Supplementary Table S1) was purchased from Shanghai Generay Biotech Co., Ltd, China. The target fragment was recombined into pET28-Sumo vector and then transformed into *Escherichia coli* Rosetta DE3 competent cells for protein expression. The recombinant 6× His-Sumo-NrS-1_Polymerase coding vector was utilized as the template during the plasmid constructions of NrS-1 N300 (residues 1–300), N392 (residues 1–392), N618 (residues 1–618), CTR (residues 301–718) and all other full-length proteins with single mutation (including K453A, K525A, N526A, R555A and R556A), by overlap polymerase chain reactions. The detailed sequences of the primers used in plasmid constructions were listed in Supplementary Table S2. All the truncations or mutants were transformed into *E. coli* BL21 DE3 competent cells for protein expression. Sequences of all plasmids were confirmed by DNA sequencing.

### Protein expression and purification

All NrS-1 polymerase proteins were expressed using the same procedures. Briefly, the frozen recombinant strains were revived in Lysogeny broth (LB) medium supplemented with 50 μg/ml kanamycin at 37°C overnight. Every 10 ml revived bacterium suspension was inoculated into 1 l LB medium and cultured at 37°C with continuous shaking. Protein expression was induced at OD_600_ ≈0.6 by adding isopropyl β-d-1-thiogalacto-pyranoside (IPTG) at a final concentration of 0.2 mM. The induced cultures were then grown at 18°C for an additional 18 h. The cells were collected via centrifugation, resuspended in Buffer A (20 mM Tris pH 8.0, 500 mM NaCl, 25 mM imidazole).

All proteins were purified using the same procedures except NrS-1 CTR. The cell pellets were resuspended in Buffer A and lysed under high pressure via a JN-02C cell crusher. The supernatant was loaded onto a HisTrap™ HP column equilibrated with Buffer A, and the target protein was eluted via AKTA purifier (GE Healthcare) using elution Buffer B (20 mM Tris pH 8.0, 500 mM NaCl, 500 mM imidazole). Then ULP protease was added to remove the 6× His-SUMO-tag, and dialyzed against Buffer C (20 mM Tris pH 8.0, 500 mM NaCl). The sample was again loaded onto the HisTrap™ HP column. The target proteins were collected, concentrated and loaded onto a HiLoad 16/600 Superdex G200 column (GE Healthcare) equilibrated with Gel filtration buffer (20 mM Tris pH 8.0, 500 mM NaCl, 2 mM DTT). NrS-1 CTR was purified using similar procedures. However, Tris pH 8.0 was replaced by Caps pH 10.0 in all above buffers. Purity of all proteins was analyzed using a 15% SDS-PAGE gel and the samples were stored at −80°C until use.

### Crystallization and data collection

NrS-1 CTR proteins in Gel filtration buffer were concentrated to 10 mg/ml. The crystallization conditions were identified at 16°C using the Gryphon crystallization robot system from Art Robbins Instrument company and crystallization kits from Hampton Research company. A-form NrS-1 CTR crystals were grown in 2.8 M Sodium acetate trihydrate pH 7.0 buffer. B-form NrS-1 CTR crystals were grown in 1.6 M NaH_2_PO_4_/0.4 M K_2_HPO_4_, 0.1 M phosphate-citrate pH 4.2 buffer. To solve the phase problem, the B-form NrS-1 CTR crystals were soaked in the crystallization solution supplemented with 1 mM methylmercury chloride for 5 min.

All crystals were cryoprotected using their mother liquor supplemented with 25% glycerol and snap-frozen in liquid nitrogen. X-ray diffraction data (Supplementary Table S3) were collected on beamline BL17U and BL19U at the Shanghai Synchrotron Radiation Facility (SSRF). Data processing was carried out using the HKL2000 or HKL3000 programs (24).

### Structure determination and refinement

The phase of B-form NrS-1 CTR structure was determined by single-wavelength anomalous diffraction (SAD) method (25) using the anomalous signal of Hg atoms and the AutoSol program embedded in the phenix suite. The initial model was refined using Refmac5 program (26) of CPP4i suite (27) and used as search model to solve the A-form NrS-1 CTR structures by molecular replacement method. The model was manually built using COOT (28) and refined using either Refmac5 or phenix.refine programs (29). During refinement, 5% of randomly selected data was set aside for free R-factor cross-validation calculations. The 2*F*_o_ − *F*_c_ and *F*_o_ − *F*_c_ electron density maps were regularly calculated and used as guides for building the missing amino acids and solvent molecules with COOT. The structural refinement statistics are summarized in Supplementary Table S3.

### *In vitro* DNA binding assays

Full-length NrS-1 polymerase, NrS-1 N300, NrS-1 N392, NrS-1 N618, or NrS-1 CTR (0.1–0.6 μM) were mixed with 50 nM 5′-FAM-labeled ssDNA1 (5′- CAGTCCGAAGCGCATCCCGTTTGACCATT-3′) in binding buffer, composed of 20 mM Tris pH 8.0, 200 mM NaCl, 5% glycerol and 2 mM DTT. The total volume of the reaction system was 10 μl. The samples were incubated on ice for 1 h and then analyzed on 6% native PAGE gels with 0.5× TBE buffer. The gel was imaged using Typhoon FLA 9000. The intensities of the bands were quantified by ImageQuantTL and the data was compared using GraphPad Prism 5.

### (d)NTPase activity assay

The steady-state rates of (d)NTP hydrolysis were determined using ATPase/GTPase Activity Assay Kit (Sigma-Aldrich). Briefly, 0.25 μM of WT NrS-1 polymerase or mutant proteins was incubated with 1 mM (d)NTP in the presence or absence of 0.5 μM DNA2 (5′-CAGTCCGAAGCGCATCCCGTTTGACCATT-3′, 3′-GTCAGGCTTCGCGTAGGGCAAACTGGTAA-5′) or ssDNA1 in reaction buffer composed of 40 mM Tris pH 7.5, 80 mM NaCl, 8 mM MgAc_2_ and 1 mM EDTA. The reactions were quenched by adding malachite green reagent at various time points. The malachite green reagent formed a stable dark green complex with free phosphate ions liberated by the enzymes. The activity of the enzyme was proportional to the dark green color intensity, which was measured at 620 nm.

### Helicase assay

DNA3 used in the helicase assay was prepared by annealing a 5′-FAM-labeled 12-nt DNA (5′-FAM-CGGGATGCGCTT-3′) with a complementary 60-nt DNA (5′-TTTTTTTTTTTTTTTTTTCAGTCCGAAGCGCATCCCGTTTGACCATTTTTTTTTTTTTTT-3′). The reaction buffer was composed of 20 mM Tris pH 8.0, 100 mM NaCl, 5 mM MgCl_2_, 5 mM dCTP, 2 mM DTT and 0.1% (v/v) NP-40. The unlabelled 12-nt DNA (5′- CGGGATGCGCTT-3′, 2 μM) was added in the reaction to anneal with the unwound DNA generated through NrS-1 helicase activity. Reaction mixtures were incubated at 20°C for 30 min and then digested by 5 mg/ml Proteinase K at room temperature for 1 h. Samples were loaded onto 10% TBE polyacrylamide gel for electrophoresis. The DNA concentration was fixed at 20 nM, the concentrations of the proteins were labeled on the gel.

### *In vitro* DNA polymerization assays

Both DNA1 and DNA4 used in the polymerization assays were purchased from Shanghai Generay Biotech Co., Ltd. DNA1 is composed of ssDNA1 (5′- CAGTCCGAAGCGCATCCCGTTTGACCATT-3′) and one 5′-FAM-labeled primer (5′-FAM-CGGGATGCGC-3′). DNA4 is composed of one template strand (5′- TGTTCAGGAACTATGTAGGTTTTGCAGAATTAGACCTTCTTGCTAGGCACTGGAACCTAAACAGTCCGAAGCGCATCCCGTTTGACCATT-3′), one downstream oligo (3′-ACAAGTCCTTGATACATCCAAAACGTCTTAATCTGGAAGAACGATCCG-5′), and one 5′-FAM-labeled primer, which is identical to that of DNA1. One 10-μl reaction system, composed of 4.5 μl reaction buffer (20 mM Tris pH 8.0, 200 mM NaCl, 5% glycerol, 2 mM DTT), 0.5 μl 100 mM MgCl_2_, 2 μl 2.5 mM dNTPs, 2 μl 5 μM DNA and 1 μl 1 μM protein, was established. The reactions were quenched by the addition of 10 μl termination buffer (90% formamide, 20 mM EDTA, 0.05% bromophenol blue and 0.05% xylene blue) at various time points. 10 μl samples were loaded onto pre-warmed 18% urea sequencing gels and run for 2 h. The gel was visualized using Typhoon FLA 9000.

### Size-exclusion chromatography and electron micrographic analysis

To analyze the oligomerization state of the full-length NrS-1 polymerase, the protein samples were dialyzed against Gel filtration buffer (20 mM Tris pH 8.0, 2 mM DTT) supplemented with high concentration salt (500 mM NaCl) or low concentration salt (200 mM NaCl), respectively. The samples were then applied to Superose 6 Increase 10/300 GL column (GE Healthcare) equilibrated with the corresponding buffer. Proteins collected from the first peak of the size-exclusion column was diluted to 40 μg/ml using the same low-salt buffer. Five microliters of protein were applied to the glow-discharged 200 mesh carbon-coated copper grids (Beijing Zhongjingkeyi Technology). The samples were stained using 0.75% uranyl formate and air-dried. Data were collected on a Talos L120C transmission electron microscope equipped with a 4K × 4K CETA CCD camera (FEI). Images were recorded at a nominal magnification of ×92 000, corresponding to a pixel size of 1.58 Å per pixel. The contrast-transfer function (CTF) parameters of each micrograph were determined using CTFFIND4 (30). Single particles were picked and processed using RELION3.0 (31). 47 138 particles were picked by using LoG-based auto-picking from RELION3.0. Reference-free 2D classification was generated using RELION. May be due to the flexible linker between NrS-1 NTR and CTR, NTR appears as hot spots in some 2D class-averages. Class-averages without hot spots were used to generate *de novo* 3D model, 3D classification, and auto-refinement by RELION.

## RESULTS

### NrS-1 CTR enhances DNA binding and polymerization

**Figure 1.**
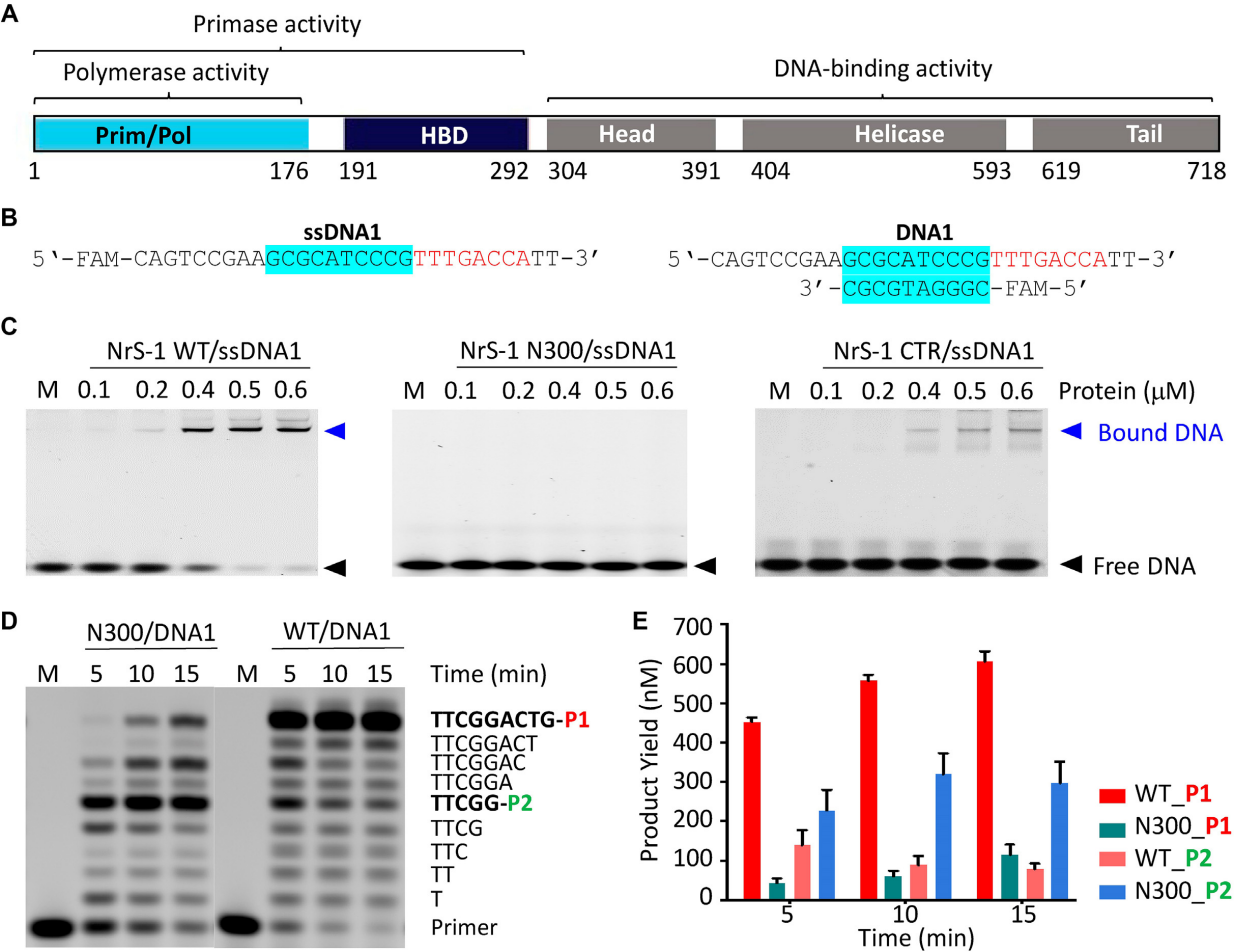
Effects of NrS-1 CTR on DNA binding and polymerization. (**A**) Domain architecture of NrS-1 polymerase. (**B**) Sequences of DNAs used in binding and polymerization assay. The recognition sequences are colored in red in both ssDNA1 and DNA1. (**C**) Comparison of *in vitro* DNA binding by the full-length NrS-1 polymerase, NrS-1 N300, and NrS-1 CTR. The DNA concentration was fixed at 50 nM. (**D**, **E**) Comparison of *in vitro* DNA polymerization catalyzed by NrS-1 N300 and the full-length WT protein of NrS-1 polymerase.

The genome of NrS-1 is linear, it's 37 159 bp in length and encodes ∼50 proteins. NrS-1 polymerase is encoded by the gene 28. Previous study showed that NrS-1 polymerase is one multidomain enzyme (Figure 1A). NrS-1 CTR (residues 301–718) was predicted to possess DNA binding affinity, however, it has not been experimentally verified. To investigate the detailed function of NrS-1 CTR, we firstly purified NrS-1 CTR and compared its DNA binding ability with those of the full-length NrS-1 polymerase and NrS-1 N300 (Figure 1B and C). Single-stranded DNA, ssDNA1 (5′-FAM-CAGTCCGAAGCGCATCCCGTTTGACCATT-3′, in which the 8-nt recognition sequence was indicated by underline), was used in the binding assay. At a concentration of 0.4 μM, the full-length NrS-1 polymerase can bind 80% ssDNA1. More than 90% and 95% ssDNA1 was bound by the full-length NrS-1 polymerase at a concentration of 0.5 and 0.6 μM, respectively. No ssDNA1 binding was observed for NrS-1 N300 under all the tested conditions. In contrast to NrS-1 N300, NrS-1 CTR could bind 10–20% ssDNA1 when the protein concentrations varied between 0.4 and 0.6 μM.

Though it was not as efficient as the full-length protein, our above binding assays showed that NrS-1 CTR plays an important role in DNA binding. NrS-1 N300 contains one PrimPol domain and one HBD domain (Figure 1A). Like the full-length protein, previous studies confirmed that NrS-1 N300 can support the DNA polymerization and de novo primer synthesis activities (21). However, might be due to its weak DNA binding ability, higher concentration of NrS-1 N300 was used. To further investigate the function of NrS-1 CTR, we compared the *in vitro* DNA polymerization activities of the full-length NrS-1 polymerase and NrS-1 N300 at the same concentration. DNA1, which is composed of one 5′-FAM-labelled primer (5′-FAM-CGGGATGCGC-3′) and ssDNA1 (Figure 1B), was used in the polymerization assays. The protein and DNA concentrations are 0.2 and 1.0 μM, respectively. As depicted in Figure 1D and -E, NrS-1 N300 could catalyze the DNA polymerization reaction toward DNA1. However, it mainly produced short run-off products, especially those with dTTCGG incorporated (named P2 hereafter). Some fully-extended products (primer with 9-nt TTCGGACTG incorporated, which was referred to as P1 hereafter) were generated, but the yield was very low. At the reaction time of 15 min, only ∼100 nM P1 product was produced. Compared to NrS-1 N300, the full-length protein was much more efficient in DNA polymerization. At the reaction time of 5 min, it produced 400 nM P1 product; and, more than 600 nM P1 product was generated at the reaction time of 15 min. These observations indicated that NrS-1 CTR is important for the catalytic efficiency of NrS-1 polymerase.

### NrS-1 CTR contains one helicase domain

**Figure 2.**
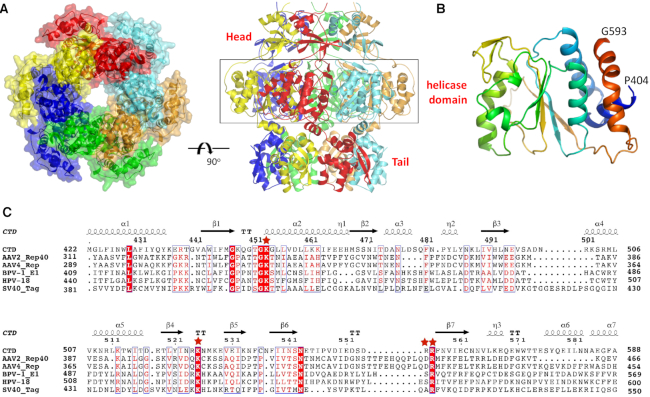
Structure of NrS-1 CTR. (**A**) Hexameric ring-shaped structure formed by NrS-1 CTR. (**B**) Cartoon view showing the overall fold of the helicase domain of NrS-1 CTR. (**C**) Sequence alignment of the helicase domains of NrS-1 polymerase and other helicases. The conserved catalytic Lys residue and Arg finger residues are indicated by asterisks.

To unravel the molecular basis underlying the function of NrS-1 CTR, we performed extensive crystallization trails. Two NrS-1 CTR structures were determined, one in A-form and another in B-form (Supplementary Table S3). The A-form crystals were grown in P2_1_2_1_2_1_ space group; per asymmetric unit contains six copies of NrS-1 CTR monomer, which were assembled into a ring-shaped architecture (Figure 2A). The B-form crystals were grown in *P*2_1_3 space group; per asymmetric unit contains two NrS-1 CTR monomers. Via symmetry operation, B-form structure can also assemble into hexameric rings, which is virtually identical to the A-form structure. Owing to its higher resolution (2.4 Å), the B-form NrS-1 CTR structure was utilized in the following structural analysis.

The ring-shaped NrS-1 CTR structure can be divided into three regions: Head, Body and Tail. The Body region is composed of residues 404–593 and has an α/β fold in nature. The Body region adopts a twisted open conformation, its central five-stranded β-sheet is flanked by α-helices from both sides (Figure 2B). Although the overall similarity is very low (∼15%), sequence alignment (Figure 2C) suggested that the Body region (referred to as NrS-1 helicase domain hereafter) belongs to helicase superfamily III, including protein E1, large T-antigen, and Rep from papillomavirus, SV40 and AAV, respectively (32). Like other helicases of superfamily III, NrS-1 helicase domain contains one Walker A motif, consisted of _447_GKQGTGKG_454_. Compared to other helicases, residues at the N-terminal half of the Walker A motif are less conserved in NrS-1 helicase domain, whereas the residues located at the C-terminal half are highly conserved. Lys453 of NrS-1 helicase domain corresponds to the catalytic Lys residue, which is critical for the function of superfamily III helicases.

### NrS-1 polymerase has multiple (d)NTPase and DNA-unwinding activities

**Figure 3.**
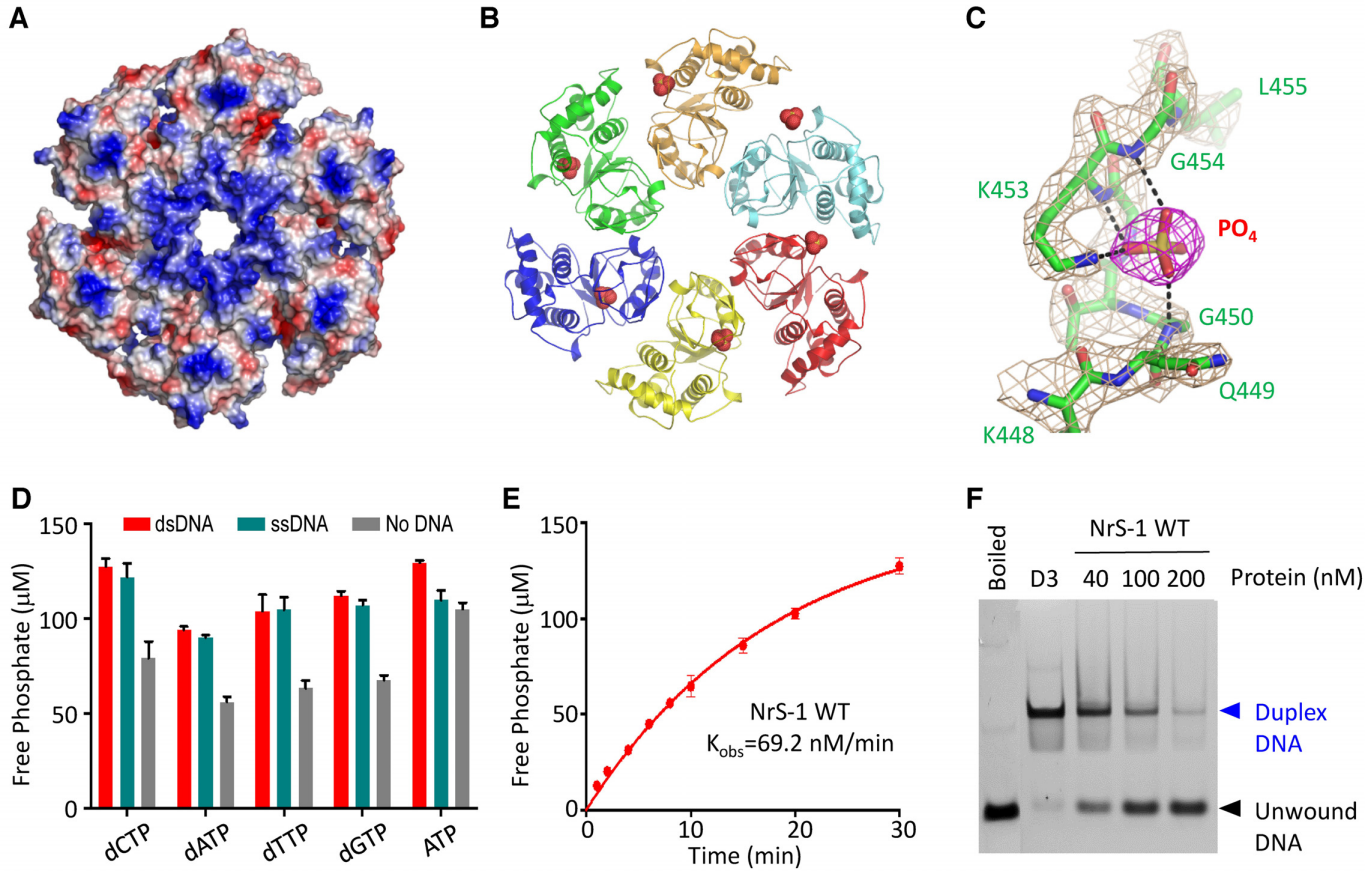
The nucleotide hydrolysis and DNA-unwinding activities of NrS-1 polymerase. (**A**) Surface presentation of the helicase domain of NrS-1 polymerase. Residues with positive charge and negative charge are colored in blue and red, respectively. (**B**) Cartoon view of the helicase domain of NrS-1 polymerase. The phosphate ions bound at the Walker A motifs are shown as spheres. (**C**) Detailed interactions between the phosphate group and the Walker A motif of NrS-1 helicase domain. The 2*F*_o_− *F*_c_ electron density maps are contoured at 1.5 sigma level. (**D**) Analysis of the nucleotide hydrolysis activity of WT NrS-1 polymerase. (**E**) Time-course analysis of the dCTPase activity of NrS-1 polymerase. (**F**) *In vitro* DNA-unwinding assays catalyzed by WT NrS-1 polymerase.

The helicase domain forms hexameric ring in both A- and B-form NrS-1 CTR structures. As depicted in Figure 3A, the interior channel of the helicase ring is highly positive in charge. Like NrS-1 CTR, ring-shaped structures have also been observed for many other proteins, including ssDNA transfer protein TrwB from *E. coli* R388 conjugative system (33) and E1 protein from bovine papillomavirus (32). The DNA translocation and/or unwinding activities of these proteins are driven by the hydrolysis of nucleotides (commonly ATP or GTP) bound at the Walker A motif. Although we failed to obtain the crystal of NrS-1 CTR complexed with nucleotides, we observed one well-defined phosphate group bound with the Walker A motif of the B-form structure (Figure 3B). The phosphate group forms four strong H-bonds (Figure 3C): one with the side chain NZ atom of Lys453 and the other three with the main chain N-atoms of Gly450, Lys453 and Gly454, respectively; the average distance is 2.8 Å.

Inspired by the structural observations, we tested whether NrS-1 polymerase possesses nucleotide hydrolysis activity using the ATPase/GTPase Activity Assay Kit (Sigma-Aldrich). As depicted in Figure 3D, NrS-1 polymerase has ATPase activity, it can efficiently release the γ-phosphate group from ATP. Interestingly, in addition to ATP, we found that NrS-1 polymerase also has hydrolysis activities toward dNTPs, the building blocks of DNAs. In the absence of DNA, the four dNTP hydrolysis activities of NrS-1 polymerase are weaker than its ATP hydrolysis activity. Both ATP and dNTP hydrolysis activities can be enhanced by DNAs. In the presence of DNA2 (5′-CAGTCCGAAGCGCATCCCGTTTGACCATT-3′, 3′-GTCAGGCTTCGCGTAGGGCAAACTGGTAA-5′) or ssDNA1, the dCTP and ATP hydrolysis activities of NrS-1 polymerase are comparable. As determined by the time-course analysis (Figure 3E), the dCTP hydrolysis rate of NrS-1 polymerase is approximately 69 nM/min in the presence of DNA2.

The above observations clearly indicated that NrS-1 polymerase possesses multiple (d)NTPase activities. Then, we wondered whether NrS-1 polymerase has DNA unwinding activity. Toward this end, we designed one overhang DNA, DNA3 (5′-TTTTTTTTTTTTTTTTTTCAGTCCGAAGCGCATCCCGTTTGACCATTTTTTTTTTTTTTT-3′ and 3′-TTCGCGTAGGGC-FAM-5′, in which the complementary sequences are indicated by underlines), and performed *in vitro* unwinding assay using NrS-1 polymerase and 5 mM dCTP. The DNA3 concentration was fixed at 20 nM. As depicted in Figure 3F, approximately 40% DNA3 was unwound by NrS-1 polymerase with a concentration of 40 nM. With a concentration of 100 nM, NrS-1 polymerase could unwind more than 65% DNA3. And, over 80% DNA3 was unwound by NrS-1 polymerase with a concentration of 200 nM. These observations clearly indicated that NrS-1 polymerase has DNA unwinding activity.

### Residues important for the helicase activity of NrS-1 polymerase

**Figure 4.**
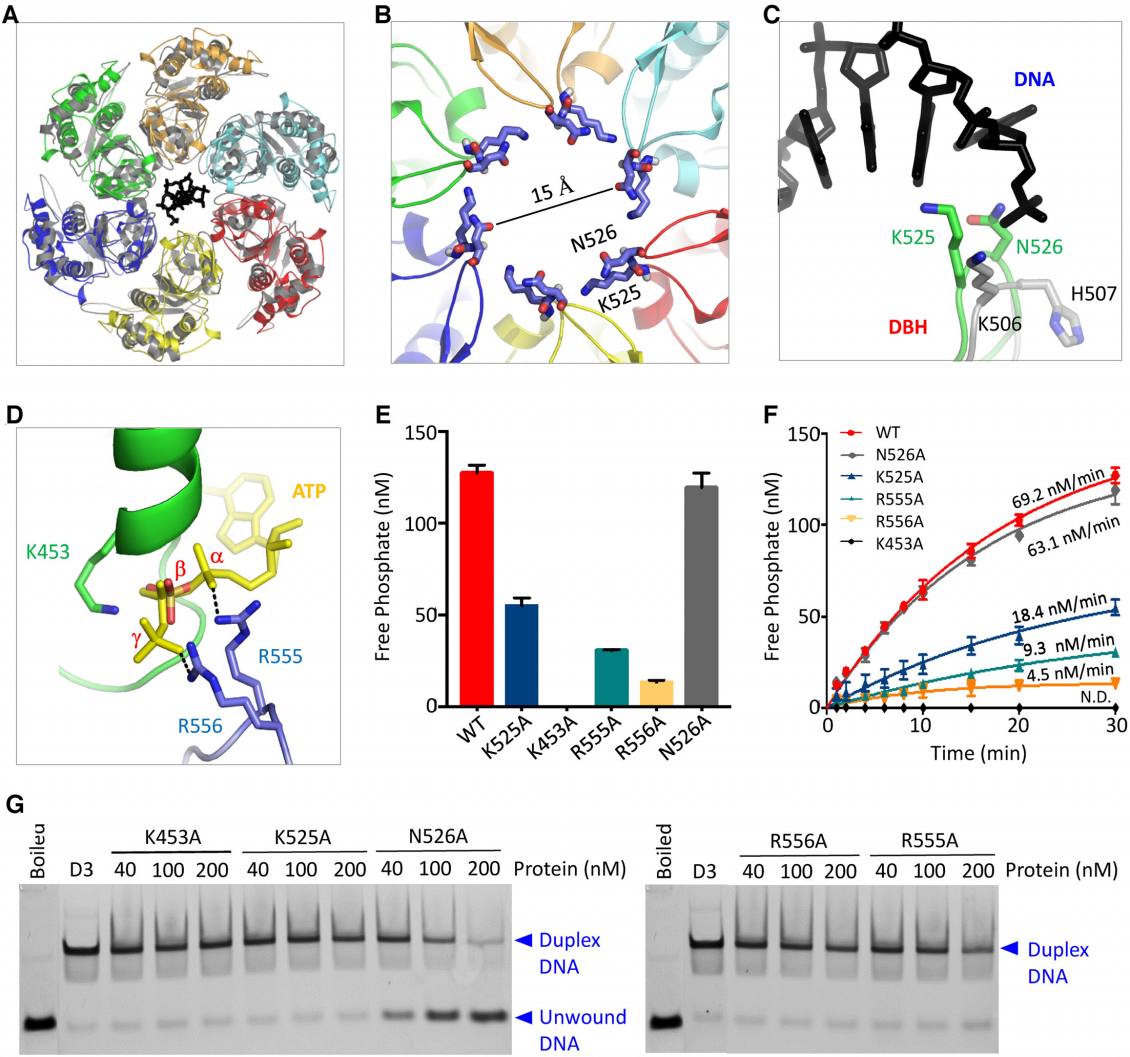
Identification of residues important for nucleotide hydrolysis and DNA unwinding activities of NrS-1 polymerase. (**A**) Superposition of the helicase domains of NrS-1 polymerase and E1 protein. E1 protein and the bound ssDNA are colored in grey and black, respectively. (**B**) ‘KN’ hairpins located at the interior channel of NrS-1 helicase domain. (**C**) Superimpose of the ‘KN’ hairpin of NrS-1 polymerase and DBH hairpin of E1 protein. The C-atoms of ‘KN’ and DBH hairpins are colored in green and grey, respectively. (**D**) Superposition showing the potential interaction between ATP and the side chains of Arg555 and Arg556 of adjacent NrS-1 polymerase. ATP was adopted from the PAN ATPase structure, which was omitted for clarity. (**E**, **F**) Comparison of the dCTPase activities of WT and mutant proteins of NrS-1 polymerase. (**G**) *In vitro* DNA-unwinding assays catalyzed by mutant proteins of NrS-1 polymerase.

To identify the residues important for the helicase activity of NrS-1 polymerase, we firstly compared the structures of NrS-1 CTR and E1 protein (PDB_ID: 2GX6). As depicted in Supplementary Figure S1A, the helicase domains of NrS-1 polymerase and E1 protein share very similar secondary structural topology. Although the flanking α-helices showed certain orientational differences, the central β-sheets could superimpose well. Like NrS-1 CTR, E1 protein also adopts one hexameric ring-shaped conformation. The arrangements of the helicase domains are similar in the NrS-1 CTR and E1 protein structures, and the diameters of their interior channels are comparable (Figure 4A). The interior channel of NrS-1 helicase domain is very narrow, it's only ∼15 Å measured at the ‘KN’ hairpin region, formed by Lys525 and Asn526. Such a narrow channel is insufficient to accommodate dsDNA, which has a diameter of around 20 Å. However, as demonstrated by the E1 protein structure, the interior channel is wide enough for binding of ssDNA. In the E1 protein structure, the ssDNA is mainly recognized by one DNA-binding hairpin (DBH), composed of Lys506 and His507. As revealed by structural comparison, the ‘KN’ hairpin of NrS-1 polymerase corresponds to DBH of E1 protein (Figure 4C and Supplementary Figure S1A), suggesting that ‘KN’ hairpin may play an important role in ssDNA binding by NrS-1 polymerase.

In addition to ssDNA, ADP molecules (the ATP hydrolysis product) were also captured in the E1 protein structure, bound with the Walker A motifs. As showed by structural superposition, the phosphate group observed in the B-form NrS-1 CTR structure corresponds to the β-phosphate group of ADP in the E1 protein structure (Supplementary Figure S1B). Besides the catalytic Lys residue from the Walker A motif, previous studies (32) suggested that the DNA translocation activity of E1 protein was also affected by elements from the adjacent subunit, such as arginine finger. The finger residues are highly conserved (Figure 2C), corresponding to Arg538 and Arg556 in E1 protein and NrS-1 polymerase, respectively. However, Arg538 did not form direct interaction with ADP in the E1 protein structure, may be due to the lack of γ-phosphate group.

An intact ATP molecule was captured in the structure of PAN ATPase (PDB_ID: 3WHK), one member of AAA+ family helicase. Different from NrS-1 polymerase and E1 protein, PAN ATPase has no DBH motif within its helicase domain (Supplementary Figure S1C), but the conformation of the Walker A motif of PAN ATPase is very similar to that of NrS-1 helicase domain (Supplementary Figure S1D). Docking the ATP molecule from PAN ATPase into NrS-1 CTR structure suggested that Arg556 may interact with the γ-phosphate group of the bound nucleotides (Figure 4D). Interestingly, NrS-1 polymerase contains one additional arginine residues, Arg555, prior to Arg556. The side chain of Arg555 points toward the α-phosphate group of the bound nucleotides, suggesting that Arg555 may also affect the (d)NTPase activity of NrS-1 polymerase.

To investigate the functional importance of above residues, we constructed five single-point mutants of NrS-1 polymerase, including K453A, K525A, N526A, R555A, and R556A, and performed dCTP hydrolysis assays. As depicted in Figure 4E and F, substitution of Lys453 by Ala (for K453A mutant) completely abolished the dCTPase activity of the protein. The dCTP hydrolysis rates of K525A, R555A, and R556A mutants are 18.4, 9.3 and 4.5 nM/min, which are 3.8-, 7.4- and 15.4-fold weaker than that of WT protein, respectively. Different from other mutants, N526A mutant showed similar dCTP hydrolysis activity to that of WT protein. To test whether the nucleotide hydrolysis activity is required for DNA unwinding by NrS-1 polymerase, we performed DNA unwinding assay using the five mutant proteins (Figure 4G). Like WT protein, N526A mutant could efficiently unwind DNA3, whereas no obvious DNA3-unwinding activity was observed for all other four mutants.

### Correlation between the helicase and polymerization activities of NrS-1 polymerase

**Figure 5.**
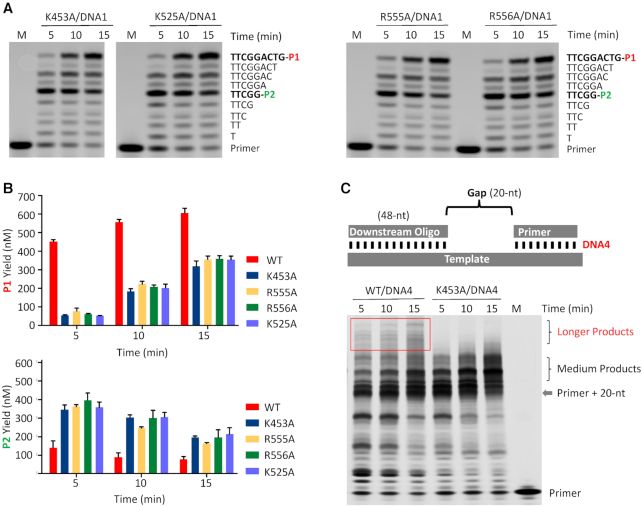
Effects of key residue mutation on DNA polymerization activity of NrS-1 polymerase. (**A**) *In vitro* DNA polymerization assays catalyzed by mutant proteins of NrS-1 polymerase. (**B**) Comparison of P1 and P2 products generated by WT and mutant proteins of NrS-1 polymerase. (**C**) *In vitro* DNA polymerization assays catalyzed by WT and K453A mutant of NrS-1 polymerase.

Both previous and our present studies showed that NrS-1 CTR can enhance the DNA polymerization activity of NrS-1 polymerase (Figure 1D). To test whether such enhancement is correlated with the helicase activity, we performed *in vitro* polymerization assays using DNA1 and NrS-1 polymerase mutants with impaired helicase activity, including K453A, K525A, R555A and R556A. As depicted in Figure 5A, the four mutant proteins can catalyze DNA polymerization reaction with similar catalytic efficiencies. At the reaction time of 15 min, they all produced about 350 nM P1 and 150–200 nM P2 products (Figure 5B). Compared to that of the full-length WT protein (Figure 1D and E), the catalytic efficiencies of these mutants are significantly weaker, indicating that the helicase activity of NrS-1 polymerase plays important role in the enhancement of DNA polymerization.

To further investigate the correlation between the helicase and DNA polymerization activities of NrS-1 polymerase, we designed one gapped DNA, DNA4, and performed *in vitro* polymerization assays (Figure 5C). DNA4 is composed of one template strand, one 5′-FAM-labelled primer, and one downstream oligo, which could form 48-bp duplex with the template. Both WT NrS-1 polymerase and K453A mutant can fill up the gap, indicated by the products that are 20-nt longer than the primer. Products with medium length can be observed in the presence of either WT NrS-1 polymerase or K453A mutant, but the longer products were only generated in the presence of WT protein. These observations clearly indicated that the helicase activity of NrS-1 polymerase can increase the processivity of NrS-1 polymerase.

### Characterization of the Head and Tail regions

**Figure 6.**
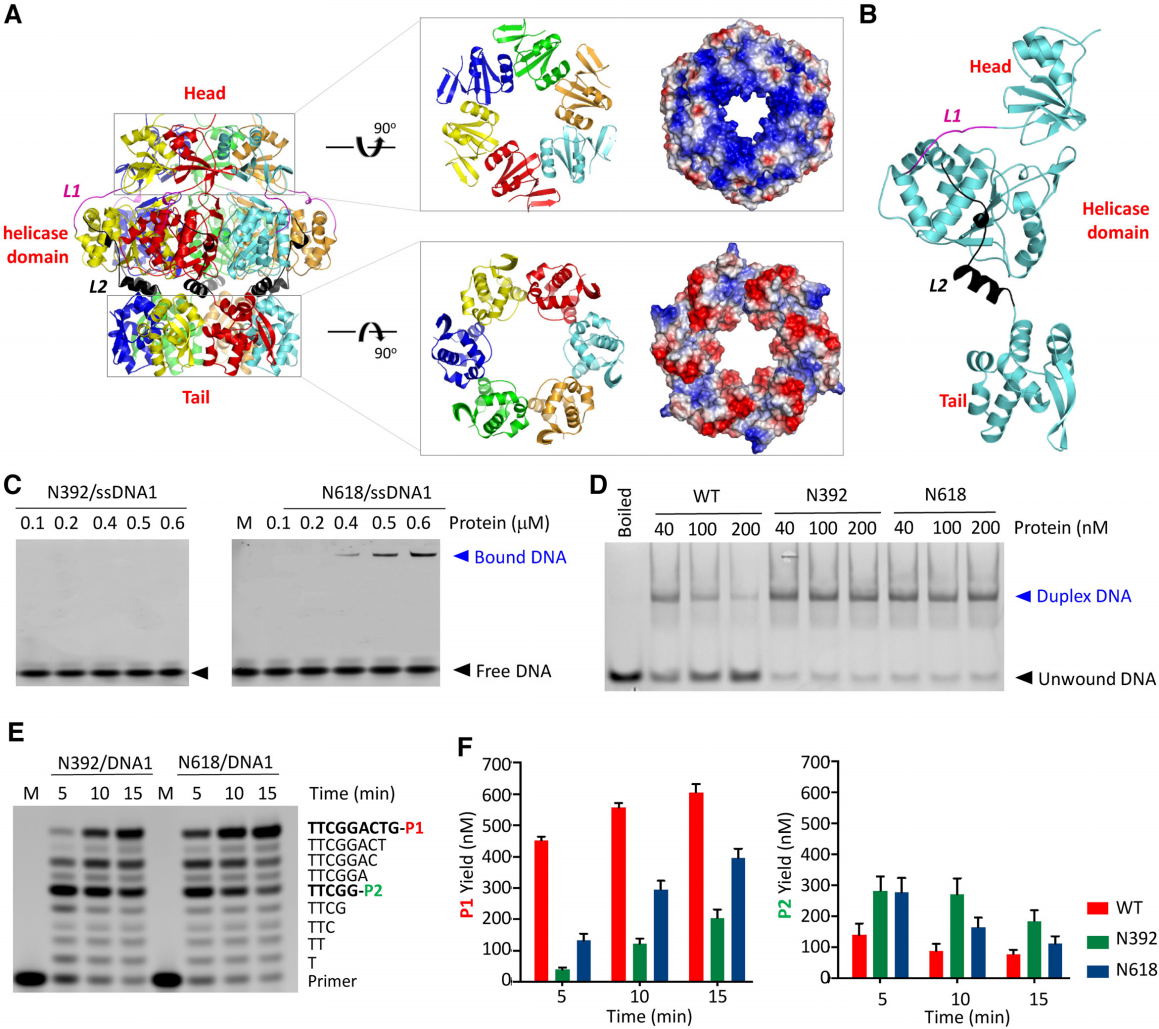
Functions of the Head and Tail regions of NrS-1 polymerase. (**A**) Assembly of NrS-1 CTR hexamer. Cartoon and surface presentations of the Head and Tail region are shown on the right. (**B**) Relative orientations of the Head, helicase domain, and Tail within the monomer of NrS-1 CTR. L1 and L2 linkers are colored in magenta and black, respectively. (**C**) *In vitro* DNA binding by truncated NrS-1 N392 and NrS-1 N618 mutants. (**D**) *In vitro* DNA-unwinding assays catalyzed by the full-length or truncated NrS-1 polymerase. (**E**) DNA polymerization assays catalyzed by NrS-1 N392 and NrS-1 N618. (**F**) Comparison of P1 and P2 products generated by the full-length and truncated NrS-1 polymerase.

In NrS-1 CTR structure (Figure 6A), the helicase domains were sandwiched between the Head and Tail regions, which are 88 and 101 residues in sizes, respectively. The Head region (residues 304–391) is connected to the helicase domain through one 12-residue (_392_TPLMKVKPVKEM_403_) linker L1 (Figure 6B and Supplementary Figure S2A). The Head is of α/β fold in nature, it contains two α-helices (α1 and α2) and six β-strands (β1–β6). β1-β3 are all-antiparallel, forming one twisted β-sheet that was flanked by α1 and α2 helices on one side and by the β4–β6 β-sheet on the other side (Supplementary Figure S2B). Like the helicase domain, the Head region also forms hexameric ring (Figure 6A). The diameter of the interior channel of the Head ring is ∼20 Å; due to the presence of Lys343, Lys356 and Lys359 at the edge or surface, the interior channel of the Head ring is very positive in charge.

The Tail region of NrS-1 CTR is connected to the helicase domain through L2 linker (residues 594–618). Linker L2 is 25 residues in length; it contains one short 3_10_-helix and one α-helix (Figure 6B and Supplementary Figure 3A). Like the Head region, the Tail region is also of α/β fold in nature (Supplementary Figure S2C), it contains six α-helices (α1′-α6′) and three β-strands (β1′-β3′). Among the six α-helices, α1′ and α5′ located in the central, packed against α2′–α4′ on one side and α6′ on the other side; β1′-β3′ are all-antiparallel. Compared to the Head and helicase domain rings, the diameter (25 Å) of the Tail ring is bigger. Different from the Head and helicase domains, the interior channel of the Tail ring are very negative in charge (Figure 6C), owing to the presence of Glu646, Glu677, and Asp682 at the edge or surface.

NrS-1 helicase domain shares sequence and structural similarities with superfamily III helicases (Figure 2C and Supplementary Figure S1), however, sequence blast and Dali search programs failed to identify any structures homology to the Head or Tail regions of NrS-1 polymerase. To investigate the functions of the Head and Tail regions, we constructed two NrS-1 polymerase mutants, NrS-1 N392 and NrS-1 N618. Both the helicase domain and the Tail region were truncated in NrS-1 N392, whereas only the Tail region was deleted in NrS-1 N618. As depicted in Figure 6C, NrS-1 N392 has no visible ssDNA1-binding ability. With a concentration of 0.4 and 0.6 μM, NrS-1 N618 polymerase could bind 5% and 25% ssDNA1, respectively. Although it is higher than that of NrS-1 N392, the DNA-binding affinity of NrS-1 N618 is much weaker than that of the full-length WT protein (Figure 1C, left panel). Different from the full-length protein, neither NrS-1 N392 nor NrS-1 N618 possesses DNA-unwinding activity (Figure 6D).

In addition to DNA binding and unwinding, we also performed DNA polymerization assays using NrS-1 N392 and NrS-1 N618. As depicted in Figure 6E and F, the DNA polymerization activity of NrS-1 N392 is very similar to that of NrS-1 N300 (Figure 1D and E). May be due to its higher DNA-binding ability, the polymerization activity of NrS-1 N618 is higher than that of NrS-1 N392. However, compared to that of the full-length protein (Figure 1D and E), the polymerization activity of NrS-1 N618 is significantly weaker. NrS-1 CTR forms hexamer in both A- and B-form structures, however, as confirmed by the size-exclusion chromatographic analysis, NrS-1 N618 exists as a monomer (Supplementary Figure S3A). Taken together, these observations indicated that the Tail region is important for the hexamer assembly and polymerization activity of NrS-1 polymerase.

### Structural assembly of full-length NrS-1 polymerase

All our *in vitro* studies indicated that incorporation of the N- and C- termini of NrS-1 polymerase is important for efficient DNA polymerization. However, NrS-1 N300 and NrS-1 CTR exist as monomer and hexamer in the reported structures and our structures, respectively. Therefore, the structure of the full-length protein is essential to elucidate the incorporation between the N- and C- termini of NrS-1 polymerase. Unfortunately, although we performed extensive crystallization trials, the full-length protein did not produce any crystal. To unravel the basis underlying the function of NrS-1 polymerase, we analyzed the oligomerization state of the protein using size-exclusion chromatography. Under high-salt condition (20 mM Tris pH 8.0, 2 mM DTT, 500 mM NaCl), NrS-1 polymerase mainly exists as a monomer, which was eluted at the volume of 15.83 ml on a Superose 6 Increase 10/300 GL column (Supplementary Figure S3B). Although the percentage is not high, we could observe some hexamer, which was eluted at the volume of 13.5 ml. Under low-salt condition (20 mM Tris pH 8.0, 2 mM DTT, 200 mM NaCl), the percentage of the hexamer and monomer becomes comparable (Supplementary Figure S3C). Interestingly, during the purification procedure, we noticed that substitution of Lys525 by Ala (for K525A mutant) can significantly stabilize the hexamer of the protein. As depicted in Supplementary Figure S3D, more than 70% protein assembled into hexamer, even under high-salt condition.

**Figure 7.**
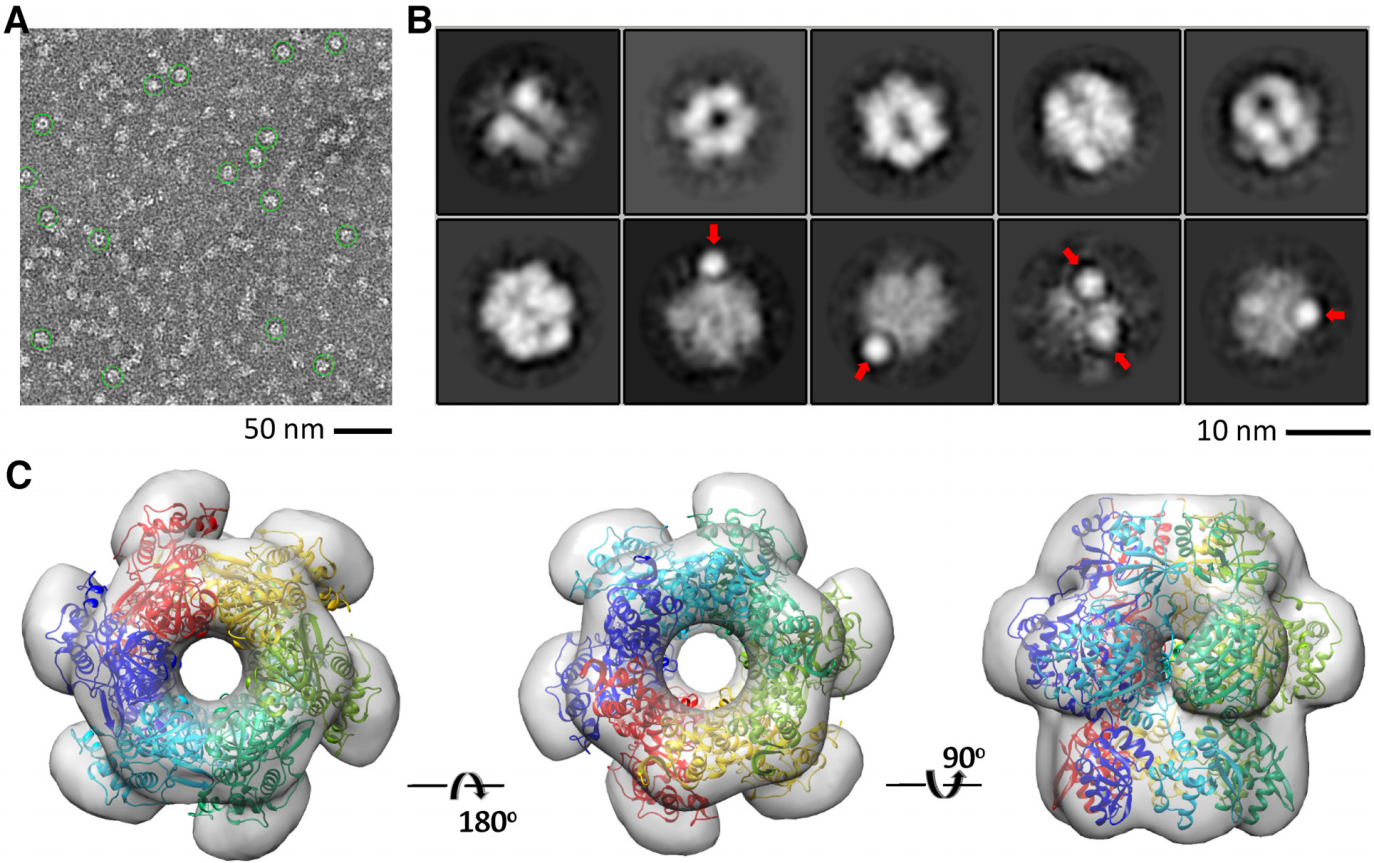
Electron microscopy analysis of full-length WT NrS-1 polymerase. (**A**) A typical negative stain CCD image of NrS-1 polymerase. (**B**) The reference-free two-dimensional class averages of NrS-1 polymerase. The maps corresponding to the N-terminus of NrS-1 polymerase are indicated by red arrows. (**C**) The electron microscopy density maps fitted with the crystal structure of NrS-1 CTR.

To further confirm the oligomerization state of NrS-1 polymerase, WT proteins collected from the first peak of the size-exclusion column were subjected to electron microscopy analysis. Many well-defined particles were observed on the negative stain CCD images (Figure 7A), which can be grouped into 10 different classes (Figure 7B). The 2D class averages clearly showed the formation of hexameric ring; the size and shape of the ring well matched with the crystal structure of NrS-1 CTR (Figure 7C). In addition to the hexameric ring, some additional electron densities were also observed on the 2D class averages (Figure 7C), which may correspond to the N-terminus of NrS-1 polymerase. As revealed by the N300 crystal structure, the linker between the Prim/Pol and HBD domains is very flexible. May be due to the flexibilities of the Prim/Pol and HBD connecting linker and the linker between the N- and C-termini of NrS-1 polymerase, only certain N-terminal regions of the hexamer could be observed. Similar to the WT protein, electron microscopy analysis showed that the K525A mutant could also assemble into ring-shaped structure (Supplementary Figure S4).

## DISCUSSION

NrS-1 is a novel phage discovered recently. Based on its morphology, NrS-1 has been assigned to the Siphoviridae family; however, the DNA sequence and genomic organization of NrS-1 are distinct from all other known Siphoviridae family phages. NrS-1 polymerase expressed in NrS-1 is one very unique PrimPol. Like other members of PrimPol, including pRN-1 and *Hs*PrimPol, NrS-1 polymerase can catalyze both DNA polymerization and primer synthesis reaction. The *de novo* primer synthesis activity of NrS-1 polymerase is largely dependent on the 8-nt recognition sequence, which is significantly longer than the sequences required by other PrimPol proteins. Due to the lack of structural information, especially NrS-1 polymerase complexed with DNA, the basis underlying recognition sequence binding by NrS-1 polymerase is not clear at present.

In addition to NrS-1 polymerase, NrS-1 also expresses one DNA helicase and one single-strand DNA binding protein (SSB), which can all enhance the DNA polymerization activity of NrS-1 polymerase (21). It was believed that NrS-1 polymerase, DNA helicase, and SSB form one replisome, which may involve in the self-replication of NrS-1 genome. DNA primase, polymerase, helicase, and SSB are all the common components of the replisome. In certain species, some components are harbored in the same polypeptide, such as the bifunctional primase-helicase in T7 bacteriophage (34). Although it was reported as PrimPol, our studies clearly indicated that NrS-1 polymerase is actually a trifunctional protein with primer synthesis, DNA polymerization, and helicase activities.

NrS-1 CTR can enhance the DNA polymerization activity of NrS-1 polymerase. As unraveled by our crystal structures, NrS-1 CTR forms one hexameric ring-shaped structure. NrS-1 CTR structure is very unique, its Head and Tail regions have no similarity to any reported protein structures. The helicase domain of NrS-1 polymerase is homology to AAA+ family helicases, however different from many other members of AAA+ family, we found that the helicase activity of NrS-1 polymerase is not strictly ATP-dependent. As showed by our *in vitro* DNA-unwinding assays, the helicase activity of NrS-1 polymerase can be supported by dCTP (Figure 3F). In addition to dCTP, NrS-1 polymerase can also hydrolyze dATP, dGTP, and dTTP (Figure 3D), indicating that these dNTPs can all function as cofactors for the helicase activity of NrS-1 polymerase. Some helicases can use dNTP to provide energy for their unwinding and/or translocation action, such as dTTP for T7 helicase (35); however, helicases with broad dNTP usage is not very common. The ability to utilize various dNTP may offer NrS-1 polymerase with more flexibility in DNA unwinding and translocation. No helicase domain is present in *Hs*PrimPol, whereas, previous studies suggested that pRN1 and other archaeal PrimPol proteins also contain one helicase domain in their C-terminal regions. It is worth to investigate whether these helicase domains form hexameric ring-shaped structure in the future.

The whole NrS-1 genome contains two copies of the 8-nt recognition sequence of NrS-1 polymerase, one in the plus strand and the other in the minus strand; both copies are in the center of the genome. Though they may serve as the origin for NrS-1 genome replication catalyzed by NrS-1 polymerase, these recognition sites are unavailable to most helicases, which require a region of ssDNA for entry. Removal of the C-terminal region of NrS-1 polymerase diminished the enhancement effect of the phage-encoded DNA helicase, suggesting that the function of the helicase domain of NrS-1 polymerase is distinct from that of the DNA helicase. NrS-1 polymerase shares both sequence and structure similarities with AAA+ family helicases, including papillomavirus E1 and SV40 Tag. Via melting of the helix and binding onto a single-stranded region, E1 and Tag can initiate dsDNA unwinding. As revealed by our size-exclusion chromatograph (Supplementary Figure S3), NrS-1 polymerase can undergo a dynamic conformational change from monomer to hexamer. Though it needs to be further confirmed, we believed that the helicase domain of NrS-1 polymerase may play certain role in dsDNA unwinding and affect the replication of NrS-1 genome.

It was believed that life originated in the ocean (36). Recent genomic analysis of marine phages has revealed the greatest gene and protein diversities on earth (37,38). NrS-1 is the first identified phage that can infect *Epsilonproteobacteria*, which is one of the predominant primary producers in the deep-sea hydrothermal vent ecosystems. NrS-1 polymerase is the only DNA polymerase encoded by NrS-1 genome. The polymerization activity of NrS-1 polymerase is strongly correlated with its helicase activity. Most likely, there are many uncharacterized marine phages existing in the ocean, which are related to NrS-1. In addition to NrS-1 polymerase, our study may also help us identify and understand the functions of multidomain polymerases expressed by NrS-1 related phages.

## DATA AVAILABILITY

Structure factors and coordinates have been deposited in the Protein Data Bank under accession codes 6LRB and 6K9C for the A-form and B-form NrS-1 CTR structures, respectively.

## Supplementary Material

gkaa071_Supplemental_File
