# Supplementary material for: Structural studies reveal a ring-shaped architecture of deep-sea vent phage NrS-1 polymerase
Source: Nucleic Acids Res. 2020 Feb 4;48(6):3343–55. doi: 10.1093/nar/gkaa071 (PMC7102993; doi:10.1093/nar/gkaa071)
Supplement: gkaa071_Supplemental_File [file gkaa071_supplemental_file.pdf]

# Supplementary Data

# For

# Structural studies reveal a ring-shaped architecture of deep-sea vent phage NrS-1 polymerase

Xi Chen[1,2], Shichen Su[2], Yiqing Chen[1], Yanqing Gao[1], Yangyang Li[1], Zhiwei Shao[1], Yixi Zhang[1], Qiyuan Shao[1], Hehua Liu[1], Jixi Li[3], Jinbiao Ma[2], Jianhua Gan[1,*]

[1] State Key Laboratory of Genetic Engineering, Collaborative Innovation Center of Genetics and Development, Shanghai Public Health Clinical Center, School of Life Sciences, Fudan University, Shanghai 200438, China

[2]State Key Laboratory of Genetic Engineering, Collaborative Innovation Center of Genetics and Development, Department of Biochemistry, School of Life Sciences, Fudan University, Shanghai, 200438, China

3State Key Laboratory of Genetic Engineering, Collaborative Innovation Center of Genetics and Development, Department of Physiology and Biophysics, School of Life Sciences, Fudan University, 200438 Shanghai, China

***Correspondence to**: ganjhh@fudan.edu.cn

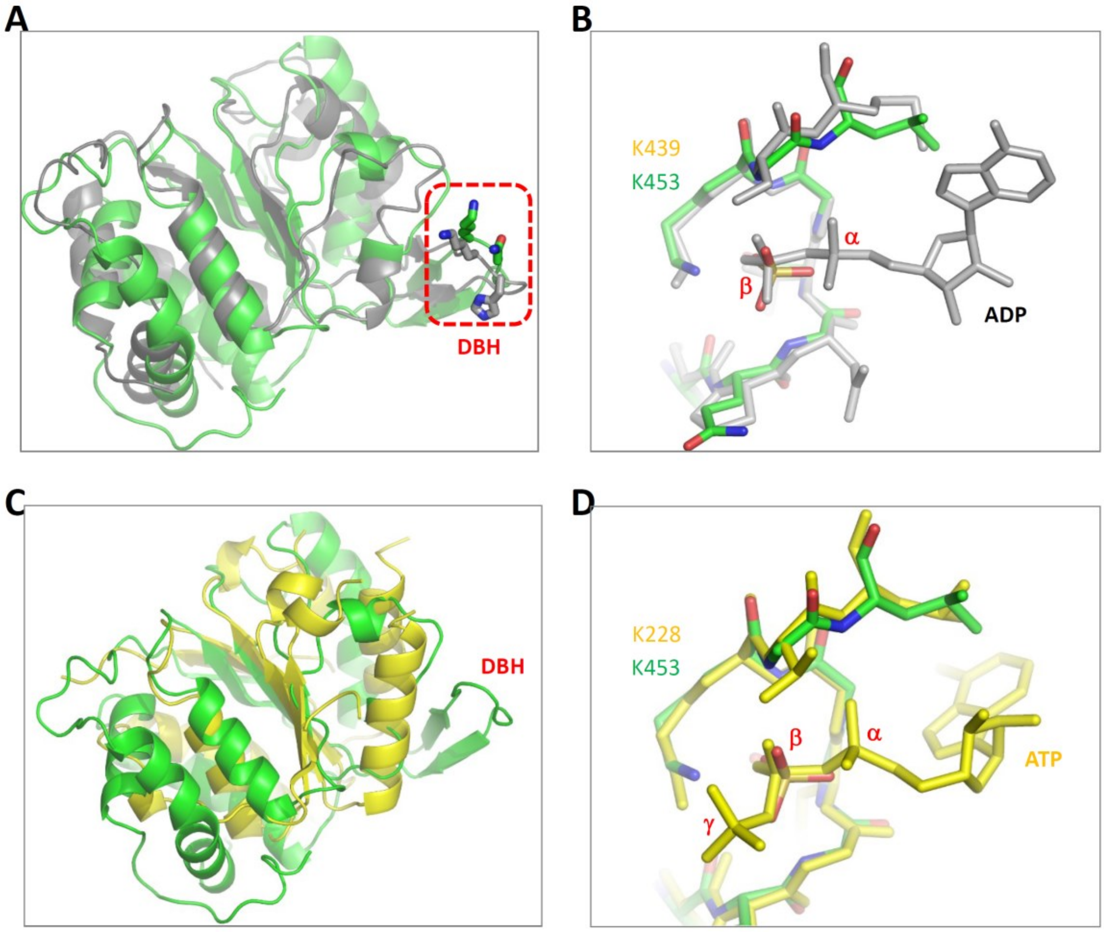


**Supplementary Figure 1.** Structural comparison between the helicase domains of NrS-1 polymerase and homologous proteins**.** (**A**) Superposition of the helicase domains of NrS-1 polymerase and E1 protein. The DNA binding hairpin was labelled as DBH and highlighted by red dashed box. (**B**) Superposition of the Walker A motifs and the bound phosphate group or ADP in the NrS-1 polymerase and E1 protein structures. (**C**) Superposition of the helicase domains of NrS-1 polymerase and PAN ATPase. (**D**) Superposition of the Walker A motifs and the bound phosphate group or ATP in the NrS-1 polymerase and PAN ATPase structures. The C-atoms of NrS-1 polymerase was colored in green, whereas the C-atoms of E1 protein and PAN ATPase were colored in grey and yellow, respectively.

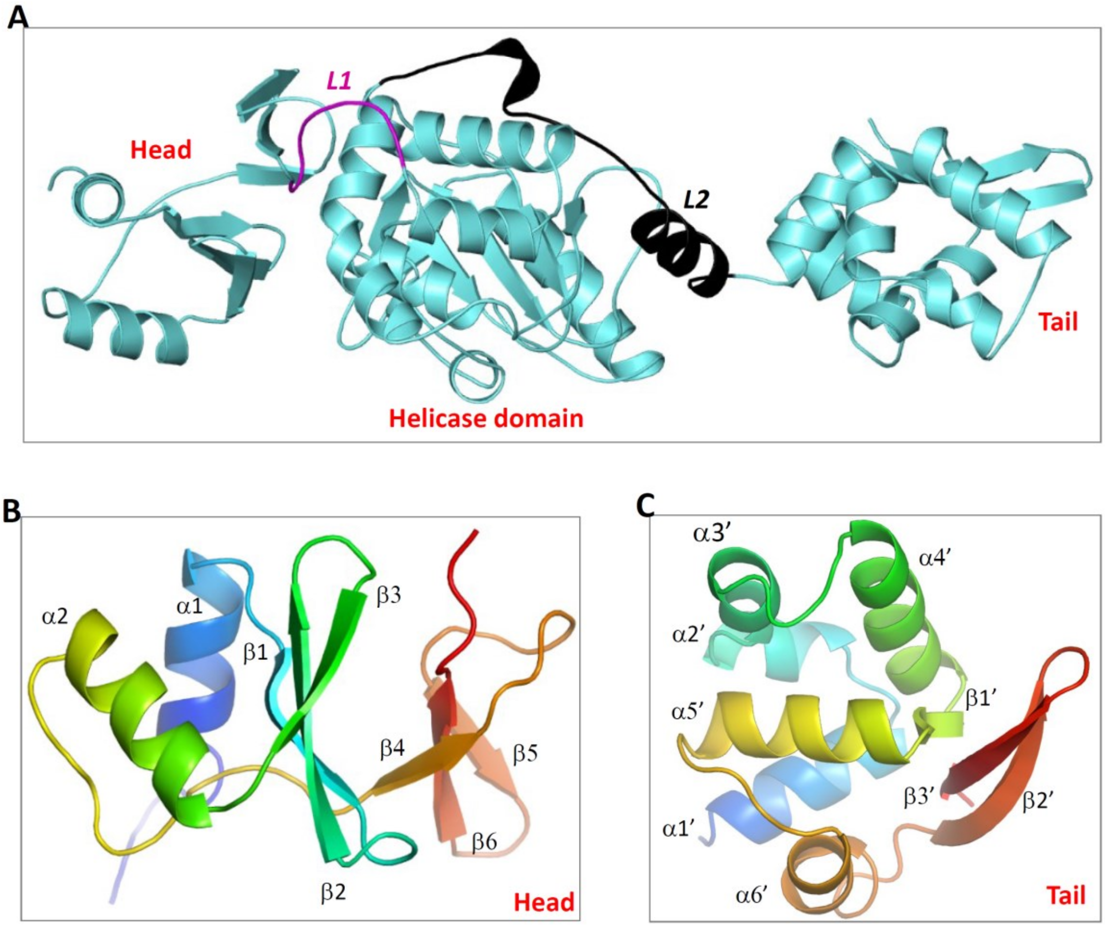


**Supplementary Figure 2.** (**A**) Cartoon view showing the overall structure of NrS-1 CTR monomer. The L1 and L2 linkers are colored in magenta and black, respectively. (**B**-**C**) Overall fold of the Head and Tail regions of NrS-1 polymerase, respectively.

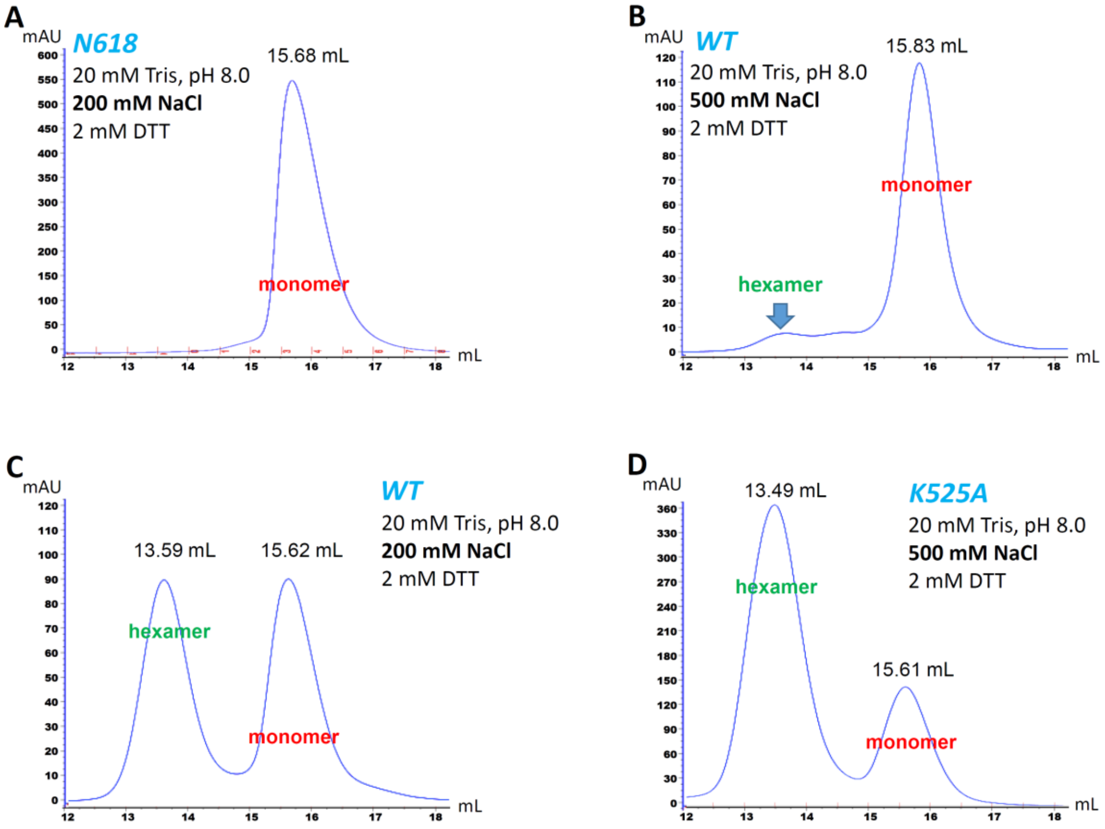


**Supplementary Figure S3.** Size-exclusion chromatographic analysis of NrS-1 polymerase. (**A**) Analysis of NrS-1 N618 protein under low-salt condition. (**B**) and (**C**) Analysis of full-length WT NrS-1 polymerase under high-salt and low-salt conditions, respectively. (**D**) Analysis of NrS-1 K525A mutant under high-salt condition. The detailed conditions are listed on the figures.

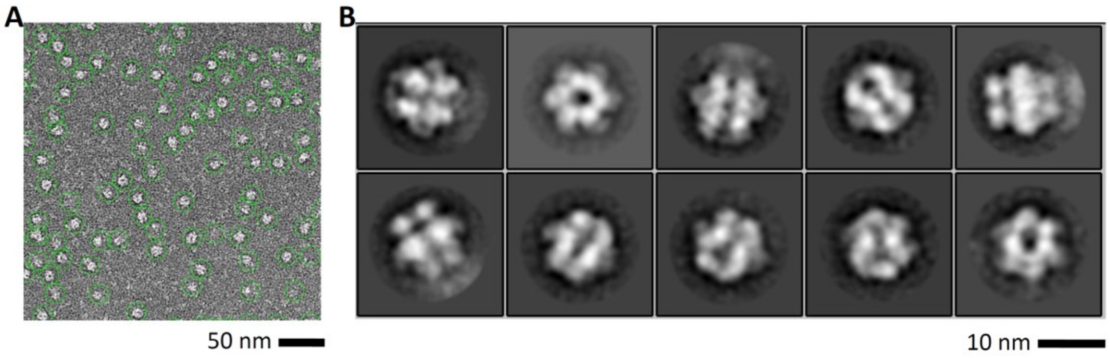


**Supplementary Figure S4.** Electron microscopy analysis of NrS-1 K525A mutant. (**A**) A typical negative stain CCD image of NrS-1 K525A mutant. (**B**) The reference-free two-dimensional class averages of NrS-1 K525A mutant.

**Supplementary Table S1**. Optimized cDNA sequences of NrS-1 polymerase.

| |
|---|
| 5′-<br>ATGATTATGGAAATTCCGGCGATTAAGGCGCTGTCCCGTTACGCGCAGTGGGTGAT<br>TTGGAAGAAAGAGCGTGACACCAAGATTCCCTATAACCCAAACAATGGCAAAAAAG<br>CGAGCTCTACGGACCCACTTGCGTGGGGAGACATCGACGAGGCGCAAGCGGGCCTG<br>GTGCGCTATGGCGCGAACGGCCTGGGCTTTGTATTAACCAAGAGCGATCCCTTCGT<br>ATTCATAGATCTGGACCATGTCCTGGACGAAAACAAGCGTGTTAAATGCGAATGGG<br>CGCGTCAGTTGTTAAAGGAAATTAAGAGTTATACCGAAATTAGCCCGAGCGGCGAT<br>GGTCTGCATGTGGTCGTCTCGGGAAAGCTGCCTGACTACATCAAGCATAAGACAAA<br>GTTCGATGATGGCAGCGCCCTGGAAGTGTATGAAAGCGGACGGTATATGACAATTA<br>CCGGCGAGGTATTTGACGGACGCGATGACATTAAGGAACTTGACCTTAGCATTCTG<br>GGAGAATTTGCGGAGCACAAGATTGAAACCAAAAATGCCCCGGTGCAGATAGAAAG<br>CGCGACCACCTTGGACGATGAGGCGATCATTGACTTGATGAAGCGGAAAGGGCAGT<br>GGCCCGATGCGCCGAAAGATGGCGATGATTGGAGCAGCCTGGATATGAGCTTTGCT<br>AACCGGCTTGCCTTCTGGTGTGGCAAGGATATCGAACGTATGGATCGTATATTTCG<br>TCAGAGCCCGCTGATGCGTCAGAAATGGGATCGTCCGACCGCGGGTAGCACCTATG<br>GCCGTATTACCCTTAAAAAGGCTTGCGACTTTGTGGACTCGGTTTATGATCCCGCG<br>CTGCGTAATGAGTCGGACTGTCCCTTTGAACCGTATAATGAAGAAGGTGGCCCTCG<br>TAACGATAAGGAGGAAAAGGACCCGCTGTGGCTTTACAAAGTGCTGTTAACGAAAG<br>GCATTGAGGTGTGGTTTGATATTAAGCTGGAAAAGTATGGAATAAAGCGTAATAAT<br>CGTGTGGATTACATCGCGAAATCCAGTCTGCAGCAAATTGTGTTTGAAATCATAGG<br>CAAAACCCCCAAAAACATAGCGGTACCAACCTATATTGGTGCATATGAACCGAGCA<br>AACCGGAGAAGTGGGAAGAAGAGGGCATCAAATATATCAATTTGTTTAAGCCGACC<br>CCGTTAATGAAAGTAAAACCGGTGAAAGAAATGCCTGAAATCGTGAAAAATCTGCT<br>GCTGAATTTATTCGATTATGATGCGAAAAGCATGGGCCTGTTCATAAATTGGTTGG<br>CGTTCATTTATCAGTATAAAGAACGGACCGGGGTGGCGTGGATTTTCATGGGCAAA<br>CAGGGCACCGGCAAAGGCCTGCTGGTTGATCTTTTAAAGAAGATATTCGAAGAGCA<br>CATGAGTAGCAATATTACCGACGCGAATCTGGATTCGCAGTTCAACCCGTATCTGT<br>ATAACAAACTGATTGTGCATCTGAATGAGGTTAGCGCGGATAACCGGAAGAGCCGT<br>ATGCTGGTGAAGAATCGCCTGAAGACCTGGATCACTGATGAGACGCTGTACATAAA<br>TCGCAAGAACATGAAGGAAGTGGAGATCAAAAACTTTTGTAATTTCATCATTAATA<br>GCAATGAGACCATACCGGTGGATATAGAGGATAGCGATCGTCGTTTTAATGTGATT<br>GAATGCAATAACGTGTTGAAGGAGCAGGAATGGTGGACCACCGAGAGCTACCAAGA<br>AATACTGAATAACGCGGAAGGTTTCGCTAAGTACCTTGCGGGCATTAAAGTGGACC<br>GTTCAAAAGTGAATGAAGTGGTGATGAGCGAAAAAAAGAAAGCTATTGTGGAAACG<br>ACTGAATCAGTCCTGAAACAGATAGCAAAAGCCCTGACGGATCGTGACATAGAATG<br>GTTTCTGGATAATGGCCTGGAGGGCGTGGTGGAGAAGAATATTGTAAATGACTTTC<br>AATGGGAGGAGCTTCAGGAAGCGATTACCACCGGCGTGATCCCTAATAAGTATCTG<br>ATGATAATAGTGGAACAAATCCTGGGCGACAGCAAGACCATTACTTGGATTAAGCG<br>CAACATTATCACACCGTATCAGGTGGGCGAGACCACGGTGGTGAAAATGGCGGGAA<br>AACCTATCCGTGCCATAGTAGTGGGC-3′ |

**Supplementary Table S2**. Sequences of primers used for NrS-1 mutant construction.

| Name | Sequence (5'-3') [a] |
|---|---|
| WT-F | GGATCCATGATTATGGAAATTCCGGCG |
| CTR-F | GGATCCAAGGACCCGCTGTGGCTTTACAAA |
| WT-R | CGCCTCGAGCTAGCCCACTACTATGGCACG |
| N300-R | CGCCTCGAGCTACTTTTCCTCCTTATCGTTACG |
| N392-R | CGCCTCGAGCTAGGTCGGCTTAAACAAATTGAT |
| N618-R | CGCCTCGAGCTATTCAGTCGTTTCCACAATAGC |
| K453A-F | AAACAGGGCACCGGCGCAGGCCTGCTGGTTGAT |
| K453A-R | ATCAACCAGCAGGCCTGCGCCGGTGCCCTGTTT |
| K525A-F | ACGCTGTACATAAATCGCGCAAACATGAAGGAAGTGGAG |
| K525A-R | CTCCACTTCCTTCATGTTTGCGCGATTTATGTACAGCGT |
| N526A-F | CTGTACATAAATCGCAAGGCCATGAAGGAAGTGGAGATC |
| N526A-R | GATCTCCACTTCCTTCATGGCCTTGCGATTTATGTACAG |
| R555A-F | GAGGATAGCGATGCACGTTTTAATGTGATTGAATGCAAT |
| R555A-R | ATTGCATTCAATCACATTAAAACGTGCATCGCTATCCTC |
| R556A-F | GAGGATAGCGATCGTGCCTTTAATGTGATTGAATGCAAT |
| R556A-R | ATTGCATTCAATCACATTAAAGGCACGATCGCTATCCTC |

[a] GGATCC and CTCGAG highlighted with underline are BamHI and XhoI recognition sequence.

**Supplementary Table S3**. Data collection and refinement statistics

| Structure<br><br>(PDB ID) | NrS-1 CTR<br>(Form A)<br>6LRB | NrS-1 CTR<br>(Form B)<br>6K9C |
|---|---|---|
| **Data collection** [a] | | |
| Space group | $P2_12_12_1$ | $P2_13$ |
| Cell parameter: | | |
| a (Å) | 150.7 | 161.1 |
| b (Å) | 150.4 | 161.1 |
| c (Å) | 149.4 | 161.1 |
| $\alpha$ (°) | 90.0 | 90.0 |
| $\beta$ (°) | 90.0 | 90.0 |
| $\gamma$ (°) | 90.0 | 90.0 |
| Wavelength (Å) | 0.9793 | 0.9793 |
| Resolution (Å) | 30.0-2.60 | 30.0-2.40 |
| Last shell (Å) | 2.69-2.60 | 2.44-2.40 |
| Completeness (%) | 97.8(93.3) | 99.7(98.1) |
| Redundancy | 6.0(3.0) | 12.5(5.1) |
| $I/\sigma(I)$ | 9.6(2.4) | 21.3(2.4) |
| Rmerge (%) | 12.0(43.5) | 8.7(49.2) |
| **Refinement** [b] | | |
| Resolution (Å) | 29.6-2.65 | 29.9-2.41 |
| $R_{work}$ (%) / $R_{free}$ (%) | 23.3/27.8 | 19.3/24.1 |
| No. of atoms | | |
| Protein | 19846 | 6709 |
| water | 20 | 81 |
| R.m.s. deviations | | |
| Bond length (Å) | 0.012 | 0.006 |
| Bond angle (°) | 1.517 | 0.846 |
| Ramachandran plot (%) | | |
| Most favored | 97.0 | 98.0 |
| Additional allowed | 2.9 | 1.8 |

[a]: Values in parentheses are for the last resolution shell.

[b]: as defined by Refmac5 program, A-form NrS-1 CTR crystal is twinned with twinning fractions of 0.382, 0.354, and 0.264 for h,k,l, -l,-h,-k, and k,-l,-h, respectively.
